# The prognostic value of D-dimer levels in metastatic osteosarcoma patients treated with second-line chemotherapy

**DOI:** 10.18632/oncotarget.11571

**Published:** 2016-08-24

**Authors:** Yujing Huang, Bangjian Liu, Yuanjue Sun, Jianjun Zhang, Yang Yao, Aina He

**Affiliations:** ^1^ Department of Oncology, Affiliated Sixth People's Hospital, Shanghai Jiaotong University, 200233 Shanghai, People's Republic of China; ^2^ Department of Neurology, Affiliated Sixth People's Hospital, Shanghai Jiaotong University, 200233 Shanghai, People's Republic of China

**Keywords:** D-dimer, osteosarcoma, chemotherapy response, prognosis, second-line chemotherapy

## Abstract

We performed a retrospective analysis of 32 metastatic osteosarcoma cases to examine the prognostic value of the plasma D-dimer level. We assessed the D-dimer level before second-line chemotherapy (D1) and the D-dimer level after two cycles of second-line chemotherapy (D2). The change in D-dimer level (ΔD) was defined as D2 minus D1. The overall survival (OS) of patients with a high D1 was significantly shorter than those with a low D1 (median OS, 4.7 vs. 16.2 months, P=0.001). Similar results were observed for the D2 (median OS, 4.7 vs. 8.6 months, P=0.033). Multivariable analysis demonstrated that a high D1 (hazard ratio, 3.375; 95% confidence interval, 1.133–10.053; P=0.029) was an unfavorable independent prognostic factor. The mean D2 of 11 patients with stable disease decreased by 0.69 mg/mL compared to the D1 (P = 0.016). The mean D2 increased by 1.47 mg/mL compared to the D1 in 21 patients with progressive disease (P = 0.004). The data suggest that D-dimer may serve as a prognostic biomarker for metastatic osteosarcoma patients treated with second-line chemotherapy.

## INTRODUCTION

Osteosarcoma is the most common primary bone cancer. It has a high propensity to metastasize to the lungs [1, 2]. Although multi-disciplinary treatments including neoadjuvant and adjuvant chemotherapy with aggressive surgical resection have improved the 5-year survival rate (approximately 70%) [1, 3], the treatment options are still unsatisfactory in osteosarcoma patients with metastasis [4]. Surgical resection has been shown to prolong the survival of patients with pulmonary metastases [5, 6]. However, for patients with multiple metastases, chemotherapy is the main strategy. The role of second-line chemotherapy is not well defined and a standard regimen has not been defined [7]. Biomarkers could enable identification of osteosarcoma patients who are likely to benefit from chemotherapy and facilitate selection of the appropriate treatment. However, markers that could predict second-line chemotherapy response in osteosarcoma patients with metastasis have not been elucidated.

Coagulation products have been associated with tumor growth, angiogenesis, and metastasis [8, 9]. D-dimer is a fibrin degradation product, which is produced when cross-linked fibrin is degraded by plasmin-induced fibrinolytic activity. The D-dimer level is widely used as an assessment tool for the diagnosis and treatment of thrombosis [10]. Elevated D-dimer levels were also associated with shorter survival times in patients with malignant tumors such as breast, colorectal, prostate, lung, pancreatic, and ovarian [11–16]. D-dimer is also a biomarker for chemotherapy response in patients with advanced lung, gastric, colorectal cancer, and serous ovarian cancer [17–20]. However, the clinical significance of D-dimer levels in metastatic osteosarcoma is not yet clear.

At our institute, we have observed elevated D-dimer levels in patients with metastatic osteosarcoma even in the absence of thrombotic episodes. In this retrospective study, we evaluated the role of D-dimer as a prognostic and predictive biomarker in patients with metastatic osteosarcoma who received second-line chemotherapy.

## RESULTS

### Patient characteristics

This study consisted of 32 osteosarcoma patients for whom we had complete clinical data. There were 18 male and 24 female patients. The baseline characteristics of the patients are shown in Table 1. The median age was 16 years. Most of the tumors (90.6%) were located in the extremities. A total of 71.9% of the patients had a Karnofsky Performance Scale (KPS) score ≥80. Four patients had pathological fractures and eight had local recurrence.

**Table 1:** Baseline characteristics of the patients

Characteristic	
Gender	
Female	14 (43.8)
Male	18 (56.2)
Age/year	
<18	17 (53.1)
≥18	15 (46.9)
Tumor site	
Extremities	29 (90.6)
Non-extremities	3 (9.4)
KPS	
≥80	23 (71.9)
≥ 70	9 (28.1)
Recurrence	
Yes	8 (25.0)
No	24 (75.0)
Pathological fracture	
Yes	4 (12.5)
No	28 (87.5)

Data are presented as percentages.

### D-dimer as a prognostic marker of survival

**Figure 1:**
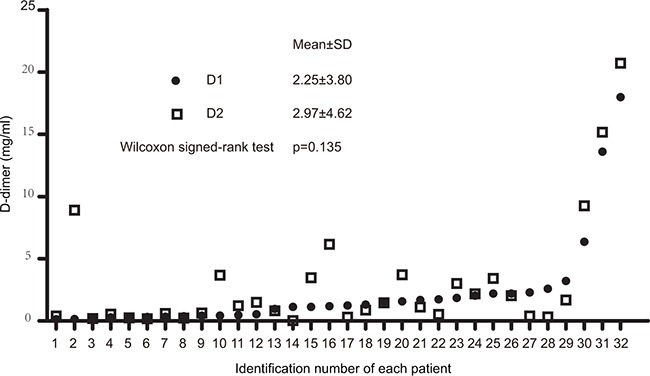
Dot plot of D-dimer levels before and after two cycles of second-line chemotherapy D1, D-dimer level before second-line chemotherapy; D2, D-dimer level after two cycles of second-line chemotherapy.

**Figure 2:**
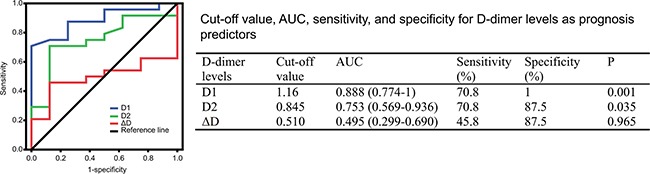
ROC curves of D-dimer levels to predict prognosis ROC, receiver operating characteristic; AUC, the area under the curve; D1, D-dimer level before second-line chemotherapy; D2, D-dimer level after two cycles of second-line chemotherapy; ΔD, change in D-dimer level(D2 minus D1).

The plasma D-dimer level before second-line chemotherapy (D1) and the D-dimer level after two cycles of second-line chemotherapy (D2) in 32 patients with metastatic osteosarcoma are shown in Figure 1. The median D1, D2, and the difference in D-dimer levels (ΔD) were 1.22(range 0.13–17.99) mg/mL, 1.17(range 0.02–20.71) mg/mL, and 0.18(range −2.26–8.75) mg/mL, respectively. Receiver operating characteristic (ROC) analyses were then performed (Figure 2). The optimal D-dimer threshold was obtained when the Youden index was maximal. The optimal cut-off values for D1, D2, and ΔD were 1.16 mg/mL (Youden index, 0.708), 0.845 mg/mL (Youden index, 0.583), and 0.51 mg/mL (Youden index, 0.333), respectively. Patients were divided into low and high groups based on these cut-off values. The patient numbers in the high D1, D2, and ΔD groups were 17 (53.1%), 18 (56.3%), and 12 (37.5%), respectively.

**Figure 3:**
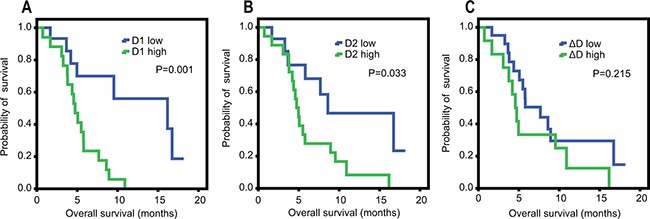
Kaplan Meier survival curves for overall survival according to D-dimer levels **A.** D1, D-dimer level before second-line chemotherapy; **B.** D2, D-dimer level after two cycles of second-line chemotherapy; **C.** ΔD, change in D-dimer level (D2 minus D1).

The median overall survival (OS) time of all patients was 5.3 (range 0.7–18.1) months. The survival curve indicated that the OS of patients with a high D1 was significantly shorter than those with a low D1 (median OS, 4.7 vs. 16.2 months, P=0.001). Similar results were observed in the D2 (median OS, 4.7 vs. 8.6 months, P=0.033). However, no differences were observed between the high ΔD and low ΔD groups (median OS, 4.6 vs. 7.7 months, P=0.215) (Figure 3).

The results of the univariable and multivariable analyses are shown in Table 2. The univariable analysis indicated that KPS (P=0.012), D1 (P=0.003), and D2 (P=0.040) were significantly associated with OS. Multivariable analysis of these factors demonstrated that a high D1 (hazard ratio [HR], 3.375; 95% confidence interval [CI], 1.133–10.053; P=0.029) was an independent unfavorable prognostic factor.

**Table 2:** Univariable and multivariable Cox proportional hazard regression analyses of overall survival

Factor	Univariable analysis		Multivariable analysis	
	HR(95%CI)	P	HR(95%CI)	P
Gender		0.088		
Female	Reference			
Male	2.136 (0.893–5.110)			
Age/year		0.936		
<18	Reference			
≥ 18	0.968 (0.431–2.172)			
Tumor site		0.274		
Extremities	Reference			
Non-extremities	0.438 (0.100–1.920)			
KPS		0.012		0.093
≥ 80	Reference		Reference	
≤70	3.390 (1.311–8.765)		2.295 (0.870–6.057)	
Pathological fracture		0.361		
No	Reference			
Yes	1.802 (0.510–6.363)			
Recurrence		0.216		
No	Reference			
Yes	0.534 (0.98–1.442)			
Chemotherapy response		0.546		
PR+SD	Reference			
PD	1.319 (0.536–3.244)			
D1		0.003		0.029
Low	Reference		Reference	
High	4.835 (1.735–13.479)		3.375 (1.133–10.053)	
D2		0.040		0.207
Low	Reference		Reference	
High	2.677 (1.044–6.868)		1.892 (0.703–5.094)	
ΔD		0.220		
Low	Reference			
High	1.682 (0.733–3.863)			
AKP		0.711		
Normal (<126 U/L)	Reference			
High	1.262(0.369–4.313)			
LDH		0.995		
Normal (<618 U/L)	Reference			
High	1.003(0.394–2.552)			

HR, hazard ratio; CI, confidence interval; SD, stable disease; PD, progressive disease; D1, D-dimer level before second-line chemotherapy; D2, D-dimer level after two cycles of second-line chemotherapy; ΔD, change in D-dimer level(D2 minus D1); AKP, alkaline phosphatase; LDH, lactate dehydrogenase.

### The relationship between D-dimer and second-line chemotherapy response

**Figure 4:**
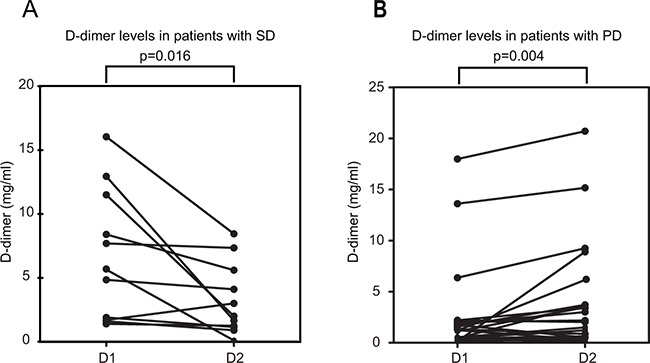
Changes in D-dimer levels in patients with metastatic osteosarcoma before and after two cycles of second-line chemotherapy D1, D-dimer level before second-line chemotherapy; D2, D-dimer level after two cycles of second-line chemotherapy; **A.** stable disease (SD); **B.** progressive disease (PD).

No differences were observed in the D1 and D2 (P =0.135) (Figure 1). We next evaluated the relationship between ΔD and treatment response. The mean D2 in11 patients with stable disease (SD) decreased by 0.69 mg/mL compared to the D1 (P = 0.016) (Figure 4 and Table 3). The mean D2 increased by 1.47 mg/mL compared to the D1 in 21 patients with progressive disease (PD) (P = 0.004). These results suggested that the D-dimer level may serve as a predictive biomarker for chemotherapy response.

**Table 3:** Differences in D-dimer levels in patients with PD and SD

Response (n=32)	D1	D2	p
PD (n=21)	2.72±4.60	4.19 ± 5.33	0.004
SD (n=11)	1.34 ± 1.02	0.65 ± 0.56	0.016
p	0.858	0.003	

SD, stable disease; PD, progressive disease; D1, D-dimer level before second-line chemotherapy; D2, D-dimer level after two cycles of second-line chemotherapy.

**Figure 5:**
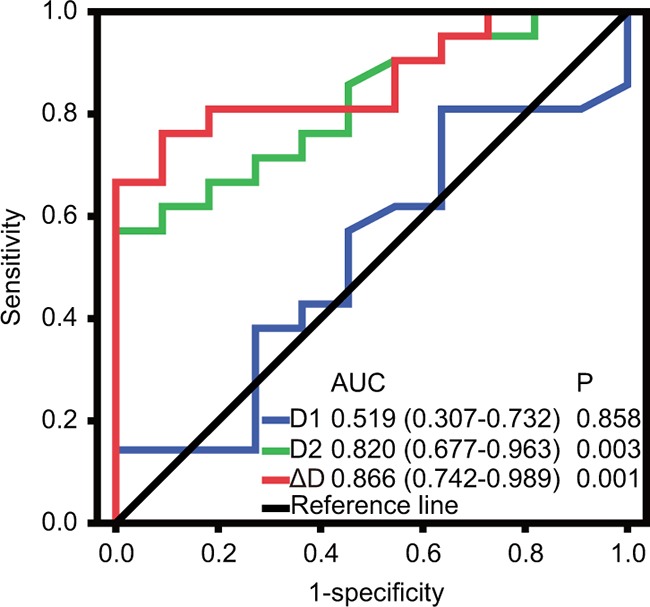
Comparison of the area under the ROC curve to assess whether D-dimer levels could predict chemotherapy response ROC, receiver operating characteristic; AUC, the area under the curve; D1, D-dimer level before second-line chemotherapy; D2, D-dimer level after two cycles of second-line chemotherapy; ΔD, change of D-dimer level(D2 minus D1).

We compared the abilities of the D1, D2, and ΔD to discriminate between responders and non-responders using ROC curves. The area under the curve (AUC) of the D1 was 0.519 (95% CI, 0.307–0.732; P =0.858). The AUC of the D2 was 0.820 (95% CI, 0.677–0.963; P =0.003). The AUC of the ΔD was 0.786 (95% CI, 0.631–0.941; P =0.009). Finally, the AUC was significantly larger for the D2 and ΔD compared to the D1 (Figure 5).

## DISCUSSION

Metastasis involves multiple tumor-host interactions. Cancer cells dissociate from the primary tumor, migrate into the circulation, attach to the vasculature, invade the surrounding tissue, and establish a new blood supply at the metastatic site [19, 21]. Cross-linked fibrin in the extracellular matrix increases the metastatic potential by serving as a stable scaffold for endothelial cell migration during angiogenesis and invasion. It also protects the cells from natural-killer cells [22–24]. Fibrin remodeling plays a crucial role in metastatic progression [25, 26]. Indeed, a reduction in pulmonary micrometastases was observed in fibrinogen-deficient and plasminogen knockout mice compared to wild-type [27–29].

D-dimer is generated by the action of factor XIIIa on fibrin monomers and polymers, and when the endogenous fibrinolytic system degrades cross-linked fibrin [30]. D-dimer consists of two identical subunits derived from two fibrin proteins. It is the final fragment generated by plasmin-mediated degradation of cross-linked fibrin. The D-dimer level is elevated in situations of enhanced fibrin formation and fibrinolysis [31]. Tumor cells can alter the balance between the coagulation, anticoagulation, and fibrinolytic systems through multiple mechanisms. This can result in hypercoagulation [32, 33]. D-dimer levels were significantly higher in cancer patients compared to healthy controls, associated with patient prognosis, and even serving as a biomarker for chemotherapy response [11, 13, 17–20, 34–36].

In this retrospective study, we evaluated whether the D-dimer level was a prognostic biomarker in patients with metastatic osteosarcoma. Our results showed that a high D1 and D2 were associated with an adverse prognosis. Univariable analysis indicated that the KPS, D1, and D2 were associated with OS. Multivariable analysis demonstrated that a high D1 was an independent unfavorable prognostic factor. The mean D2 in 11 patients with SD was significantly lower than the mean D1. In contrast, in 21 patients with PD, the mean D2 was significantly higher than the mean D1. These data indicated that the D-dimer level could be a useful prognostic marker in metastatic osteosarcoma.

Patients with PD had a significantly higher D2 than patients with SD. Patients with SD had a lower D2 compared to patients with PD. The D2 was higher in patients with PD. These results indicated that the D1 and D2 could be useful markers of tumor response to second-line chemotherapy. However, previous studies have reported decreased D-dimer levels after chemotherapy in PD patients (not a significant difference) [17, 18]. The differences between our data and the data from previous studies could have resulted from study heterogeneity. For example, our patients were treated with second-line chemotherapy, but most of the other studies evaluated the D-dimer level in patients who were treated with first-line chemotherapy [13, 18, 20].

**Figure 6:**
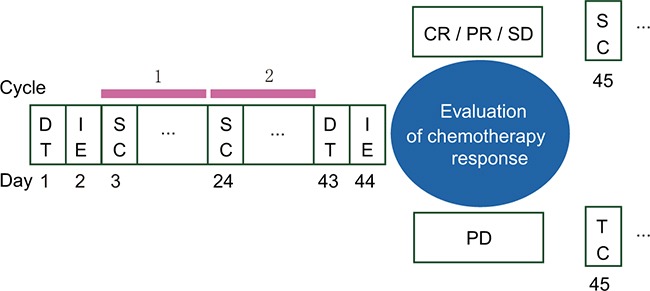
Treatment schedule DT, D-dimer test; IE, imaging examination; SC, second-line chemotherapy; TC, third-line chemotherapy; CR, complete response; PR, partial response; SD, stable disease; PD, progressive disease.

We also assessed whether the D1, D2, and ΔD could be used to discriminate between responders and non-responders using ROC curves. The AUC values were 0.519, 0.820, and 0.786 for the D1, D2, and ΔD, respectively. The AUC values for the D2 and ΔD were larger than the values for the D1, which indicated that the D2 and ΔD had a stronger association with tumor response. The D-dimer level was measured close to the start of chemotherapy and the tumor response assessed according to the treatment schedule (Figure 6). Based on our results, D-dimer has diagnostic value. It may also be a predictive factor of tumor response. To test this hypothesis, we will change the timing of the D2 measurement to right after the first cycle of second-line chemotherapy in a prospective study.

Measurement of D-dimer levels is simple and cheap compared to imaging examinations. A new model based on the D-dimer level may be particularly useful for patients in developing countries. However, our study had several limitations. First, it was a retrospective, single-institution study with a small sample size. As a consequence, it provided a lower level of evidence than a randomized controlled trial. Second, we excluded patients for whom there was incomplete clinical data. This could have resulted in selection bias. The cut-off values for the D-dimer levels were also likely biased because they were determined based on ROC analysis. Finally, heterogeneity in the treatment approaches could have affected the results. Chemotherapeutic agents produce coagulation imbalances by causing endothelial injury, decreases in coagulation factors synthesis in the liver, and/or platelet dysfunction [37, 38]. Different second-line chemotherapies could have altered D-dimer levels, which would also have biased our results.

In conclusion, our data indicate pre- and post-chemotherapy D-dimer levels are correlated with OS in patients with metastatic osteosarcoma. Moreover, the D2 is a robust predictor of survival. The D2 and ΔD were strongly associated with tumor response. These results indicate that D-dimer levels could be used to predict prognosis and treatment response in patients with metastatic osteosarcoma. Additional prospective studies are necessary to validate these findings.

## MATERIALS AND METHODS

### Patients

We retrospectively reviewed the medical records of 32 patients with osteosarcoma who were treated in our department between January 2006 and June 2015. The inclusion criteria for the primary studies were the following: (i) patients with histologically confirmed osteosarcoma; (ii) patients who received second-line chemotherapy and had available D-dimer measurements pre- and post-chemotherapy. Patients were excluded from the final analysis for the following reasons: (i) acute illness such as infection within the 2 weeks preceding D-dimer measurement; (ii) use of anticoagulants at the start of second-line chemotherapy; (iii) other primary malignancy; and (iv) incomplete data. The study was approved by the Medical Ethics Committee of the Shanghai Jiaotong University Affiliated Sixth People's Hospital.

### Data collection

Clinical data including sex, age, KPS score, tumor location, histologic type, plasma levels of alkaline phosphatase and lactate dehydrogenase, pathological fracture, response to second-line chemotherapy, and survival duration were collected. D-dimer levels were measured before and after second-line chemotherapy and the change in levels reported.

Treatment response was typically evaluated by computed tomography/magnetic resonance imaging according to the Response Evaluation Criteria in Solid Tumors [39]. Treatment responses were classified as complete response (CR), partial response (PR), PD, and SD. Only patients with SD, PR, and CR continued chemotherapy.

### Statistical analysis

All statistical analyses were performed using the SPSS statistical software (Version 19.0, IBM Corp.). D-dimer levels were presented as the mean ± standard deviation and compared using Wilcoxon signed-rank tests. OS was defined as the time from the date of treatment with second-line chemotherapy to the date of the last follow-up or death from any cause. The optimal cut-off values for the D1, D2, and ΔD were determined by ROC analysis using OS as the end-point. Kaplan-Meier analysis was performed to generate survival curves. To identify independent prognostic factors, univariable and multivariable analyses were performed using Cox regression models. The AUC was calculated and compared to evaluate the abilities of the D1, D2, and ΔD in discriminating the response to second-line chemotherapy. P values were two-sided, and a P < 0.05 was considered statistically significant.
